# Traveling Letters, No. III

**Published:** 1844-08

**Authors:** 


NEW ORLEANS, May, 1844.

*To my Colleagues:*

GENTLEMEN—In my last, a few days since, you had, I fear, rather a tedious account of some things connected with the Charity Hospital. My present will not oppress you with statistics nor perplex you with problems, two most desirable exemptions, when the mercury is aspiring to 90°. I propose in the first place to say a few words on the

### *Medical College of Louisiana.*

This institution, as we say in Kentucky, is 'picking up.' Since my visit last spring, its professors have built a new house—an edifice which will be found large enough for 150 students or more, and is planned with considerable skill. There are in it two lecture rooms, a dissecting room, a cabinet room, &c., and its exterior appearance is respectable, if not magnificent. As yet the collections of *materiel* are not as extensive as I should have expected, but the faculty are making efforts to increase them, and to engraft upon the institution a medical and philosophical reading-room. The number of pupils last year was about 80, which is an encouraging increase on all past sessions. The professors of this college seem to be left to the prosecution of their enterprize with less of public co-operation and professional sympathy, than is common in such cases. All which the State of Louisiana has done, I believe, is to allow the edifice to be built on an *acute* angle of the public rhomboid (I cannot call it a square) which could not be used for any thing else; and the city has done still less—that is to say, as far as I can hear, it has done nothing. Familiar as you are with the thought of what the city of Louisville has done for her cherished institution, you will scarcely comprehend the neglect of New Orleans; but still the difference may be accounted for. The native population of Louisville and the immigrant, are of the same kind; and, therefore, readily unite in objects designed to advance the interests and raise the character of the city. But the natives of New Orleans and the immigrants are immiscible, and, like uncombinable atoms, float among each other without coalescing. The true natives are the creoles, who take not the least interest in the school, but send their medical students to Paris, quite as much as before the transfer of Louisiana to the United States; and, as to the Anglo-American emigrants, with a small number of exceptions, they feel no interest in any thing, but the commerce which attracted them hither, to retain them till they make a fortune, and no longer. But, I have said, the school does not enjoy the sympathy, and I may add, the good will, of the profession in the city. The French physicians are not represented in it, as I think they should be, and contribute nothing to its support; and as to the American, most of them consider themselves quite as well qualified to teach as their brethren who have been chosen. Thus it is, that the professors are left to take care of themselves. Under these sinister circumstances, it is not marvellous that the school has not had a vigorous growth. But there are other causes of retardation, more permanent if not more efficient. Students of medicine are either poor or in 'moderate circumstances,' and New Orleans is an expensive place of residence; there are moreover, many alluring temptations to pleasure and dissipation; finally, it is almost destitute of literary and scientific associations, and consequently its social atmosphere is unfavorable to the healthful progress of the student. On the other hand, the opportunities for the study of anatomy, both healthy and morbid, are, or rather might be made, superior to those of any other city in the Union, and the wards of the Charity Hospital afford opportunities for clinical observation, on many, if not all forms of disease, as ample as could be desired. These elements of medical instruction will no doubt at last prevail, and build up a great school in the metropolis of the South-west.

Of the existing Faculty I cannot speak if I would, for I am not acquainted with the whole, have had opportunities of professional intercourse with but two or three, and have heard but one of them lecture.

The professor of chemistry, well known in the West by his writings on Botany and Geology, is now engaged on a very different matter. Being refiner of the mint, he has acquired possession of a vast number of counterfeit coins, and is about to publish a book of *fac similies*, by which they can be known.

The professor of the Institutes has lately published a small volume on the nervous system, which is said to evince very considerable research and acute thinking. I have not yet had time to examine it, but hope one of you will present whatever it contains of original matter to our readers. Another, the professor of materia medica, has more lately still published a large pamphlet on the transmission of yellow fever, in which he presents an array of *ex parte* facts, so imposing as to have carried conviction to the minds of some professional and many non-professional persons, who, in conformity with his recommendations, are becoming the advocates of a

*Quarantine.*

In this recommendation we find physicians who think and feel alike, on few other topics, heartily concurring. The quarantine party has three head men. Professor Carpenter, of whom I have just spoken, Dr. Luzenberg, who represents the party opposed to the college, and Dr. Beugnot, who stands at the head of the French profession. The two latter made a report to the Legislature at their last session, in favor of a quarantine, and Professor Carpenter's book was designed to act in the same quarter. Notwithstanding the facts and arguments presented by these distinguished advocates of importation, two of whom at least were educated in the theory of local origin, the Legislature refused to grant a quarantine. Whether this was right or wrong, I am not prepared to say, but I fear, that should it be attempted, such a quarantine as would be necessary to a satisfactory experiment, will not be maintained; and, certainly, no other is desirable to either party. The State of Louisiana should feel and manifest an interest in *this* quarantine. If the disease is really imported into New Orleans, it is of course propagated by commercial operations to the towns of the interior. For them to establish a quarantine against New Orleans, would greatly embarrass the business and intercourse of the people on the Lower Mississippi, and, at last, village quarantines will be found as inefficient as they will prove annoying. Nothing but a city quarantine should, therefore, be attempted. This would shackle one branch of foreign trade, but leave all the rest, and that of the interior, unobstructed. An effective quarantine below New Orleans would render any above it unnecessary; for if the disease should still appear in that city, it would prove one of two things—that the disease is of local origin, or cannot be excluded by restrictions on commerce, and, in either case, quarantines above the city would be absurd. I cannot perceive that a quarantine of the city would essentially affect its exterior commerce, which consists more in the exportation of cotton than importations from all quarters of the world taken together. If vessels from the ports whence the yellow fever is *supposed* to be introduced, were, for half the year, subjected to quarantine, the inconvenience could not be serious; as a standing regulation of that kind, would cause the chief importations to be made in the other half. Unsatisfied with every theory of the origin of yellow fever, I should be glad to see a decisive experiment on any one of them. In connexion with this subject, I may speak of the

*Medico-Chirurgical Society,*

of which Dr. Luzenberg is president. It embraces about 30 members, of whom not more than half were present on the evening of my attendance. It is regarded as a party society, and under that feeling some physicians have not been offered membership and others have declined it. The society is incorporated, and meets once a month, when papers are read, and the business common to such associations transacted. The report in favor of the *importability* of yellow fever, of which I have spoken, was made to this society, by Drs. Luzenberg and Beugnot, Dr. Mercier, a promising young Creole surgeon, dissenting. An attempt to get a vote of the society in its favor was unsuccessful; but yet they sent it to the Legislature, where, as I have stated, it failed to convince the majority that a qarantine ought to be authorised. Had it gone less ambitiously into the consideration of other diseases, and more into the proofs that *yellow fever* is imported, its effect on the minds of the honorable body, to whom it was addressed, might have been different. The society proposes, among other laudable objects, to investigate this subject by original observations, but unless its members and committees proceed in their labor, as though they had not already 'made up their minds,' I fear they will not very soon settle the question.

*Professional Brotherhood.*

Another object intended to be accomplished by this society is to unitize and harmonize the profession in New Orleans, but it is easy to perceive, that instead of working out that desirable end it is already producing the opposite. Indeed, my impression is, that from the 'nature of things' in this great city, such harmony will not show itself much before the time when the suspended materials of the Mississippi will crystalize into soluble salts, and its waters become transparent.

New Orleans is, and must continue to be, famous for individuality of character. Among its people, the divellent forces rather exceed the attractive, and are likely to remain in the ascendant. It is the residence of the many, the home of a few; and the majority of the former are little inclined to allow themselves to be drawn into any social alliances. They come hither, as I have intimated, to make or remake their fortunes, and think lightly of any thing else. Our brethren participate in the general character of the place, and many are quite unacquainted with each other. But few of them have been long in the city, and still fewer, I judge, are inclined to identify themselves with its various social and scientific interests. One of the oldest, in point of residence, is a graduate of Transylvania University of 1825 or '26; and a very large proportion are much younger in their professional age. Thus they are coming and going—some to the grave, some to other places, and some into other business. All this, as I philosophically remarked, a few sentences back, arises from the 'nature of the case,' and will continue till the city shall rear up her physicians from among her native sons; till when, no remarkable concord and co-operation are to be expected.

I have seen many things here, which would interest you and our readers, as new, but must confine myself to professional matters, of which I have not time at present to say anything more.

Your obedient servant,

